# Second-Order Effects in Lightweight Aggregate Concrete Slender Columns

**DOI:** 10.3390/ma18061312

**Published:** 2025-03-16

**Authors:** Ewelina Kołodziejczyk, Tomasz Waśniewski, Vojtěch Starý, Richard Dvořák, Martyna Rabenda

**Affiliations:** 1 Department of Concrete Structures, Lodz University of Technology, Politechniki 6, 93-590 Lodz, Poland; tomasz.wasniewski@p.lodz.pl (T.W.); martyna.rabenda@p.lodz.pl (M.R.); 2 Department of Construction Management and Economics, Faculty of Civil Engineering, Czech Technical University in Prague, Jugoslávských Partyzánů 1580/3, Praha 6-Dejvice, 160 00 Prague, Czech Republic; vojtech.stary@fsv.cvut.cz; 3 Institute of Building Testing, Faculty of Civil Engineering, Brno University of Technology, Veveri 331/95, 602 00 Brno, Czech Republic; richard.dvorak@vut.cz

**Keywords:** lightweight aggregate concrete, LWAC, reinforced concrete, RC columns, second-order effects

## Abstract

This paper covers the analysis of the behavior of columns made of LWAC (Lightweight Aggregate Concrete), which can be used as a substitute for normal-density concrete. This work presents an experimental research program focused on second-order effects in slender elements (λ = 74) made of LWAC and normal-density concrete tested under eccentric load. Elements with two ratios of longitudinal reinforcement (0.9%, 2.3%) were considered. Despite the similar compressive strengths of the concrete, the significantly different moduli of elasticity of the two types of concrete caused the lightweight concrete columns to show higher displacements, leading to their lower load bearing capacity. This disproportion was more pronounced for low longitudinal reinforcement. In the second part of this experiment, the behavior of the columns was simulated using models taken from the literature. The analysis showed that the models often do not accurately predict the elastic modulus or LWAC limit strain of concrete, so when trying to predict the behavior of this type of element accurately, these parameters should be determined experimentally.

## 1. Introduction

The constant search for alternatives to resource-limited raw materials is an ongoing process in all fields. One of the developments aligned with this trend is the search for alternatives to the typical aggregate for concrete, which is gravel [1,2,3]. The commonly considered substitute are lightweight aggregates (LWA). In some cases, they can be on natural origin (like pumice [4,5], scoria, volcanic tuff and lava ash), but to a much greater extent, artificial ones are used, produced using raw materials from minerals or industrial waste. The raw materials that are most often used in this case are expanded shale and clay (Leca, Liapor) [6,7,8] or perlite [9,10]. When it comes to the types of industrial waste, the most common are power plant slag, sintered fly ash (Certyd, Lytag and Pollytag) [11,12,13,14], granulated blast furnace slag, metallurgical pumice, fly ash cenospheres [15] and oil palm shell [16,17]. Moreover, LWA allows concrete to be produced with less density in all cases than that of traditional aggregate. The physical properties of different LWAs are summarized in [2]. The justifications for using this group of materials are varied; the main ones can be considered to be combating the issues of reducing the dead weight of a structure, improving insulation performance and fire resistance, and intending to use waste materials per the policy of sustainable development [2].

From the point of view of structural design, the most important aspects are the physical and mechanical characteristics of the concrete produced from LWA. Regarding these properties, lightweight aggregate concrete (LWAC) is classified based on its density in the oven-dry state. According to standard EN-1992-1-1 [18], concrete is considered lightweight if its density in the oven-dry state does not exceed 2000 kg/m^3^. Modern concrete technology makes it possible to produce relatively strong structural concrete for densities as low as 1200 kg/m^3^ [19,20]. Regarding the abovementioned reasons for using LWAC, any reduction in density provides benefits in terms of dead weight and thermal properties. Still, it also has other consequences, such as increased deformation due to the low elastic modulus. Moreover, we must also consider another characteristic of LWAC, which is its high brittleness.

These properties could prove to be of concern in structures sensitive to excessive displacement and those in which we expect the ductile performance of elements and the redistribution of internal forces. 

The latter issues were analyzed in [21], where a study of two-span beams was presented, and it was shown that the redistribution of forces in LWAC (1840–1850 kg/m^3^, f_c_ = 50 MPa) beams was possible, albeit to a slightly lesser extent than in concrete of typical density (2250–2280 kg/m^3^, f_c_ = 40–45 MPa, NWAC). The topics of ductility were also addressed in [22], where tests of over-reinforced concrete beams subjected to four-point bending were presented. LWAC had a mass density of 1800 kg/m^3^, with a compressive strength of 35 MPa. The results showed that providing stronger links and/or fibers allows brittle behavior to be avoided.

Several previous works have dealt with LWAC column capacity and deformability analysis, mainly in the context of the confinement effect. These studies mainly covered short elements. Various strengthening methods were considered, such as hemp fiber-reinforced rope polymer (FRRP) wraps [23], CFRP or BFRP jackets [24] (in this case, square columns were tested), transverse reinforcement [25,26,27], steel spirals and fibers [28]. The results in all cases confirmed that the beneficial effects of confinement were possible to achieve, which resulted in columns exhibiting higher deformability and, in some cases, higher load carrying capacity. More slender elements were analyzed in [29], where a study of columns made of LWAC with a density of 1920 kg/m^3^ and a strength of 60 MPa was presented. The results showed that introducing concrete confinement through strong transverse reinforcement positively affected the elements’ behavior in terms of ductility and post-critical bearing capacity. This paper also analyzed second-order effects and presented the verification of the results obtained by the nominal curvature method included in [18]. However, the tested columns were still relatively short (λ = 21), making it impossible to relate the results to elements with significant second-order effects. In conclusion, it can be said that concrete confinement has a positive effect on the load bearing capacity and deformability of confined compression members made of LWAC, but there is still a need to extend these observations to members with greater slenderness.

Second-order effects, as mentioned above, are related to the slenderness of elements, but equally important in this regard is their stiffness, which is dependent on the elastic modulus of concrete. This important material characteristic is one of the key differences that distinguishes LWAC from so-called normal-density concrete (NWAC), and it has a much lower value for the former. The second feature attributed to LWAC is its brittleness, which is related to its low ultimate strain at failure. This issue, too, requires further analysis in the context of its impact on the load bearing capacity and failure mode of slender columns, possibly indicating the need to improve the ductility of these types of elements. For these reasons, it is important to evaluate the behavior of slender elements made of LWAC in terms of their sensitivity to second-order effects compared to those made of concrete with typical parameters (NWAC). A nonlinear analysis of slender LWAC columns based on the concrete models in [18] was carried out by the authors of [30]. Still, due to the lack of experimental testing, the validation of the conclusions against the actual results was not included. The analysis of previous LWAC tests presented in this paper showed that the elastic modulus estimated according to [18] was often overestimated, raising concerns about overestimating the load carrying capacity of slender columns. The conclusions confirmed the great sensitivity of the results of the load bearing capacity of slender LWAC elements to density-dependent material properties. Therefore, the formulas for the modulus of elasticity referring to LWAC and, as a result, the peak strain, because it has almost linear behavior, should be considered only the first approximation because the modulus is significantly affected by various variables, such as moisture and the type of aggregate. It was claimed that slender columns should be considered as elements where deflections are of great importance, and in this case, the modulus of elasticity should be determined experimentally.

This work will focus on the experimental evaluation of the effects of the characteristics of two different concretes (lightweight and normal) of the same strength on the load bearing capacity and deformability of slender reinforced concrete columns and attempt to create a computational model to simulate their behavior. In the aforementioned work, a theoretical analysis was already carried out, but at that stage, the authors did not have experimental studies of such elements, so it was not possible to compare the results obtained from modeling with the actual behavior of the columns. Moreover, there were concerns about overestimating the load capacity of the elements. These considerations prompted the authors to carry out the research presented below.

## 2. Materials and Methods

### 2.1. Experimental Program

This research was focused on second-order effects in slender reinforced LWAC columns. The results were planned to be compared with those obtained for the elements of typical NWAC. The type of LWAC was chosen based on the availability of lightweight aggregate in the Polish market. The aggregate used was expanded clay, available under the trade name Liapor (Czech Republic). This study included eight elements, four of each type of concrete (LWAC and NWAC). The compressive strength of concrete in both cases was planned to be the same. This study aimed to investigate elements with dimensions close to those typical for practical applications. The columns were designed as slender columns (λ = 74) with a rectangular cross-section. It was planned to test four types of elements that differed in terms of their longitudinal reinforcement ratio and concrete type, and two identical specimens were prepared for each type to test the repeatability of the results. The columns were designed according to Eurocode 2 [18].

### 2.2. Elements

The dimensions of the elements were the same in all cases. The length of the columns was 3000 mm. The height and width of the cross-section were 140 and 250 mm, respectively.

**Figure 1:**
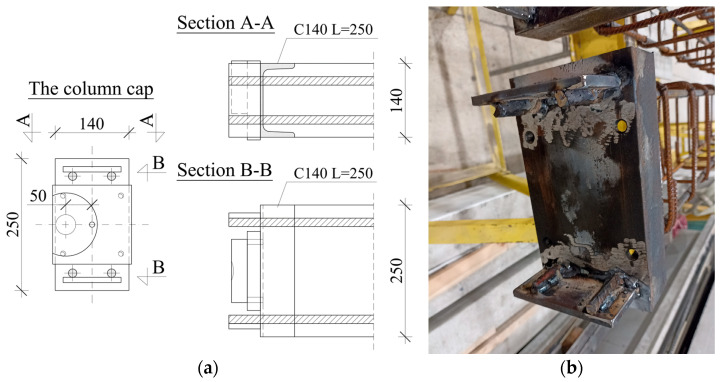
Column cap arrangement—details (**a**) and a photo (**b**).

The column ends were covered with caps made of the C140 profile. Threaded holes were bored in the end caps, serving as a place to screw down 25 mm thick steel plates. Additional 30 mm steel plates were attached to the first plates, with a socket to place a dynamometer or support bolt. The socket was located according to the assumed eccentricity of the load value. The details of the column cap are presented in Figure 1.

**Figure 2:**
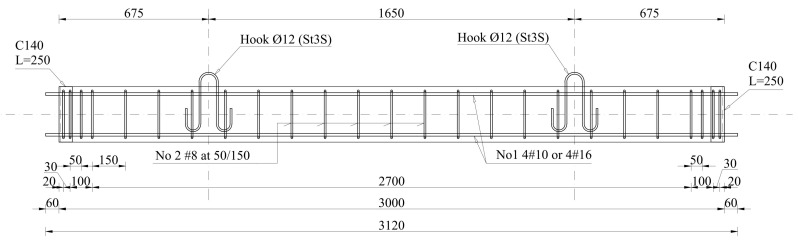
Column reinforcement.

The reinforcement consisted of 4 ribbed longitudinal bars and stirrups. There were two different longitudinal reinforcement ratios (ρ_L_) considered—2.3% (4 bars of 16 mm diameter) and 0.9% (4 bars of 10 mm diameter). The longitudinal bars had mechanical anchors (welded plates) at column caps. The diameter of the bars was included in the name of the elements to distinguish them. The transverse reinforcement was made of 8 mm rebar. The stirrup spacing was set at 150 mm along most of the element length with 150 mm long strengthened zones at the ends, where it was reduced to 50 mm to avoid local concrete crushing. The arrangement of the column reinforcement is presented in Figure 2.

The elements were made in a horizontal position in steel formworks. During the first three days after pouring concrete, columns and samples were sprinkled with water.

Detailed information regarding the columns is presented in Table 1.

### 2.3. Materials

The presented research used two types of concrete: LWAC and typical NWAC. The compressive strength of concrete was to be about 60 MPa for cylinder specimens, and the same value was planned to be achieved in both cases.

**Figure 3:**
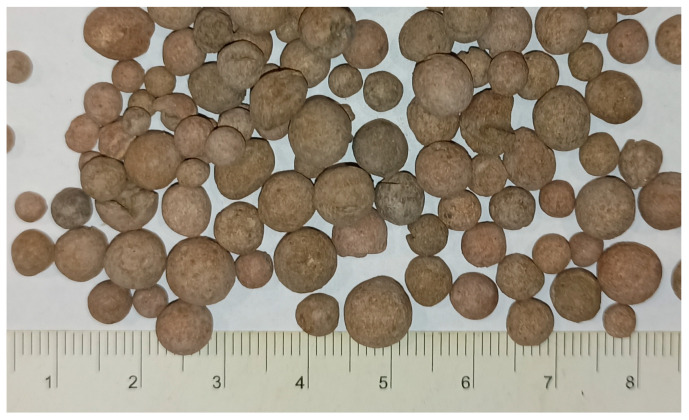
The lightweight aggregate Liapor.

The LWAC mixture was prepared at the Laboratory of the Department of Concrete Structures (LUT). It included lightweight course aggregate Liapor 8 (2–8 mm)/800 (Figure 3) with a declared crushing resistance of 15 MPa. The fine aggregate was typical sand (0–2 mm). Table 2 presents more detailed mix proportions for LWAC.

Normal-weight concrete was delivered from an external commercial source. In this case, all NWAC columns were cast from the same batch. The mix proportions for NWAC are presented in Table 2.

Cylindrical and cubic samples were prepared during each casting procedure. It was planned to determine the compressive strength f_c_, f_c,cube_, modulus of elasticity E_c_ and splitting tensile strength f_ct,sp._ The tests were carried out approximately 100–130 days after the casting. The sets of samples assigned to a given column were tested on the same days as the column tests were conducted. The LWAC and NWAC properties obtained in the tests are shown in Table 3.

**Figure 4:**
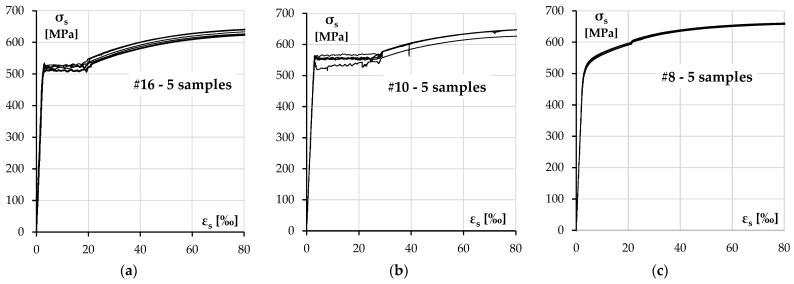
A reinforcing steel stress–strain diagram: (**a**) #16 rebars, (**b**) #10 rebars and (**c**) #8 rebars.

There were three diameters of ribbed bars used in the columns—#10 and #16 mm bars for longitudinal reinforcement and #8 for transverse reinforcement. The nominal yield strength in each case was declared to be 500 MPa. The test results for each size of the reinforcement are presented in Figure 4 and Table 4.

### 2.4. Test Stand and Measurement Instrumentation

**Figure 5:**
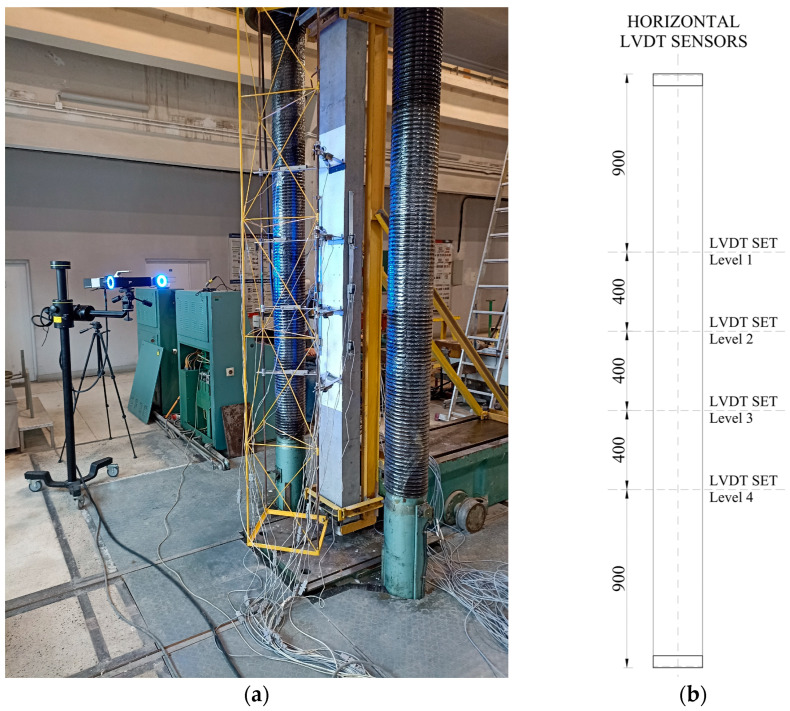
Overall view of test stand (**a**) and horizontal LVDT placement plan (**b**).

The columns were loaded vertically in a Class 1.0 hydraulic press of 6000 kN capacity. Figure 5a presents a view of the test stand. The top and bottom ends of the element were secured against excessive movement with a steel frame visible in the photo. The elements were assumed to work in a simply supported static scheme with a constant eccentricity of the load applied through an accessory at the head. The applied eccentricity was 50 mm.

**Figure 6:**
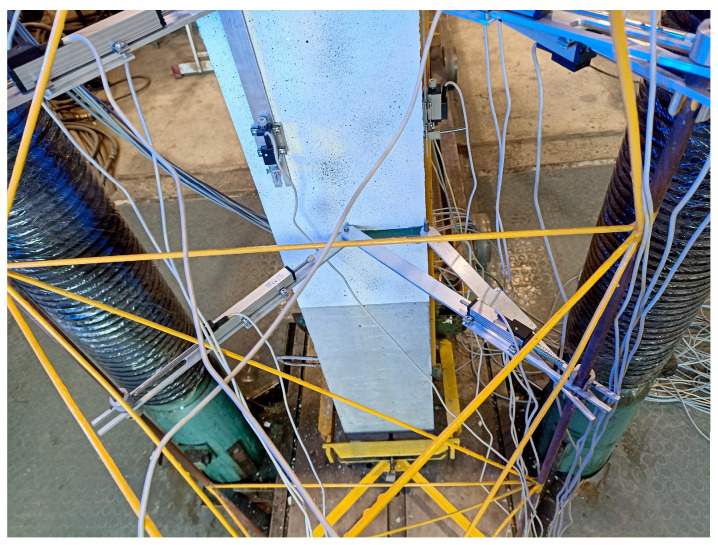
Configuration of horizontal LVDT sensors.

A steel truss was attached to the front of the stand, which allowed for the placement of LVDTs (linear variable differential transducers) for measuring horizontal displacement. The sensors were installed at four levels (see Figure 5b). The configuration of the sensors at each level is presented in Figure 6.

**Figure 7:**
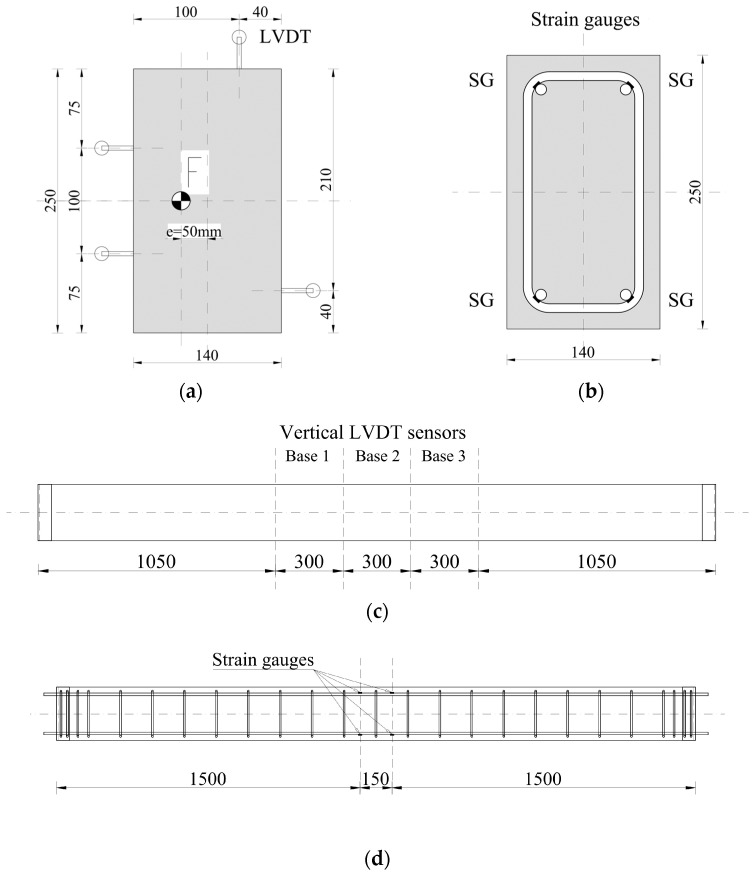
The location of the vertical LVDTs in the cross-section (**a**) and along the column (**c**). The location of the strain gauges in the cross-section (**b**) and along the bars (**d**).

LVDTs were also installed to measure vertical strain on three sides of the column at the central part of the element. Three sensors were used in a line. The base length was 300 mm. The location of the vertical transducers is presented in Figure 7a,c. Figure 7a also shows the location of the force in the section with its eccentricity.

The columns were also equipped with strain gauges to measure the longitudinal strain of the reinforcement. Strain gauges were placed on each longitudinal bar at two levels in the middle part of the column, as shown in Figure 7b,d.

In addition to traditional measuring equipment, an Aramis 3D Camera System was used to capture displacement on the front surface of the columns during the whole procedure (see Figure 5a).

### 2.5. Loading Procedure and Measurement Methods

Compression tests of the columns with constant force eccentricity were conducted more than 100 days after the casting. During the initial loading phase, the force was increased in increments of 15 kN until the elements entered the cracked state. The force at which the first phase was considered complete was estimated as 150 kN for columns reinforced with #10 bars and 180 kN for columns with #16 bars. Then, the load was withdrawn until the level was about 5 kN. In the third phase, the load was continuously increased until the element failed.

**Figure 8:**
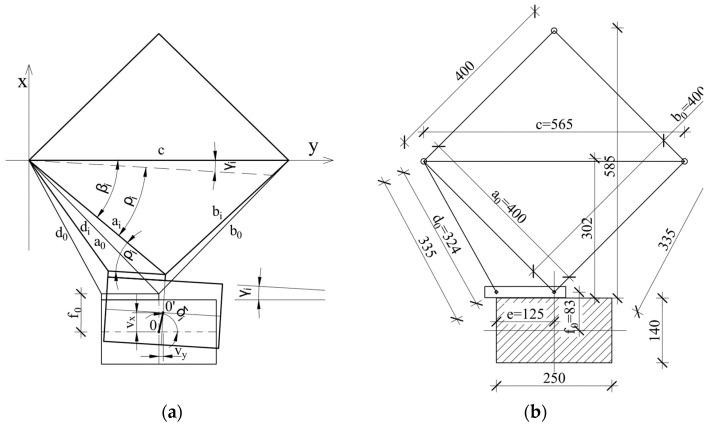
The displacement measurement system: the layout (**a**) and main dimensions (**b**).

During the test procedure, it was planned to measure the displacement of the element in the horizontal plane in both the x and y directions, as well as the rotation of the cross-section. The first direction was parallel to the force eccentricity, and the second allowed for the control of possible movements in the secondary direction. The displacement measuring system is depicted in Figure 8a,b (also see Figure 6).

The x-y coordinate system was set at one of the corners of the measurement truss (Figure 8). The displacement of the column was defined as the displacement of the center of its section, whose initial position was denoted as point 0 (x_0_, y_0_) and its subsequent positions as point 0’ (x_i_, y_i_), and the coordinates can be calculated using the following equations:

(1) $$x_{i}=a_{i}sin\left(\beta_{i}\right)+f_{0}cos(\gamma_{i}).$$

(2) $$y_{i}=a_{i}cos\left(\beta_{i}\right)-f_{0}sin(\gamma_{i})$$

They can further be determined by taking into account the following relations:

(3) $$a_{i}=a_{0}-\Delta a_{i}$$

(4) $$b_{i}=b_{0}-\Delta b_{i}$$

(5) $$d_{i}=d_{0}-\Delta d_{i}$$

(6) $$cos\left(\beta_{i}\right)=\frac{a_{i}^{2}+c^{2}-b_{i}^{2}}{2a_{i}c}$$

(7) $$cos\left(\rho_{i}\right)=\frac{a_{i}^{2}+e^{2}-d_{i}^{2}}{2a_{i}e}$$

(8) $$\gamma_{i}=\beta_{i}-\rho_{i}$$

Then, these can allow us to find the displacement of the column axis at the measured levels:

(9) $$v_{xi}=x_{i}-x_{0}$$

(10) $$v_{yi}=y_{i}-y_{0}.$$

The vertical strain of the surface of the concrete was also calculated. It was determined based on the displacement records from LVDT sensors, their base distance and their location in the cross-section (see Figure 7a).

## 3. Results

### 3.1. Experimental Results

#### 3.1.1. Failure Mode

**Figure 9:**
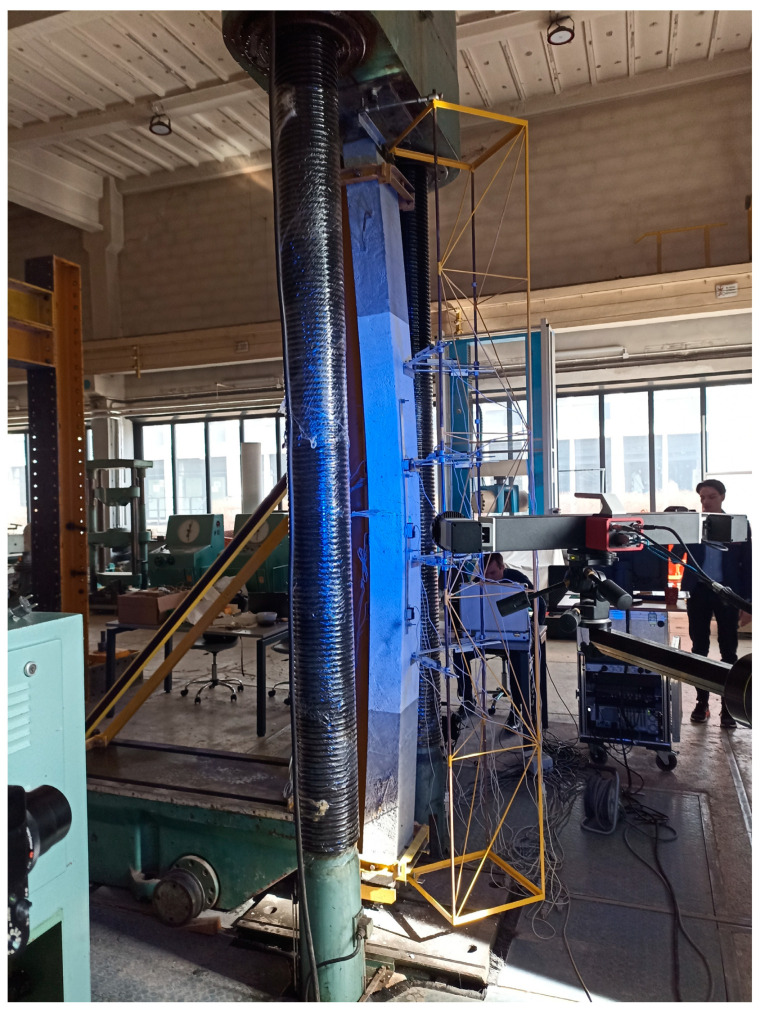
The typical buckling behavior of the columns at the maximum load (CL-16-2).

All elements displayed similar buckling behavior at the failure stage. The typical shape of the columns at the maximum load is presented in Figure 9.

**Figure 10:**
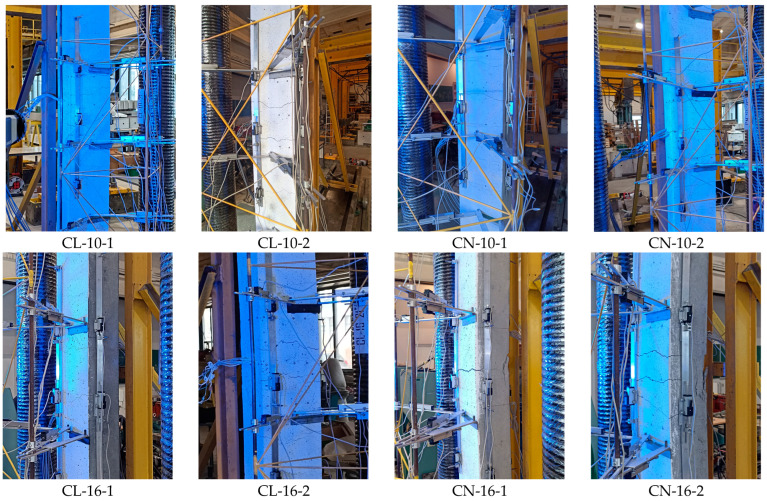
The middle part of the columns at the moment of failure.

As the load increased during the test, more cracks appeared, and deflection developed. The test was terminated when a pressure drop in the press was noted. Figure 10 shows photos of the center zones of the columns at the time of failure. In most cases, only cracks visible on the tensile surface indicated the column’s failure. For columns CN-16-1 and CN-16-2, the cracks were accompanied by crushing the concrete on the compression side of the element.

#### 3.1.2. Ultimate Load and Deflection

A summary of the test results is presented in Table 5. As detailed above, three types of columns were designed, and each came in two copies. The results of the corresponding elements were given one after the other. The displacements of the columns and the load level were monitored during the test. The table shows the value of the maximum force F_u_ and the corresponding displacement at the half-height of the column (calculated as the average value from the two sets of sensors that were located closest to the center of the element; see Figure 5b) in the buckling plane v_xu_ and the perpendicular direction v_yu_, respectively (see Figure 8).

**Figure 11:**
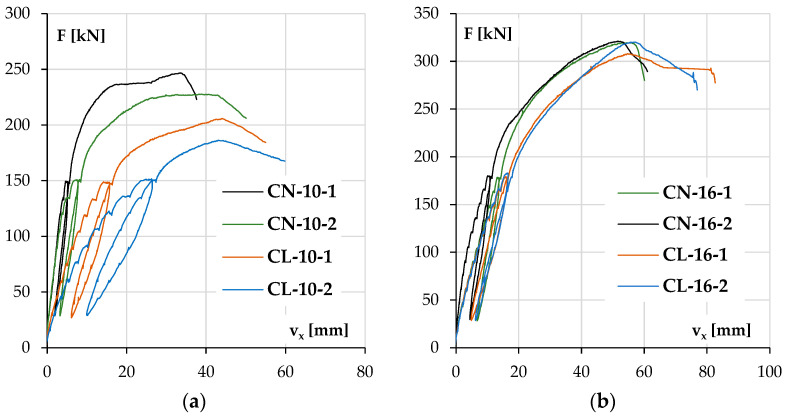
The experimental displacement v_x_ results as a function of load F for the columns with lower reinforcement ratios (**a**) and higher reinforcement ratios (**b**).

The diagrams in Figure 11a for columns with lower reinforcement ratios and Figure 11b for the stronger ones depict the development of the element deflection in the main plane (v_x_) with increasing load.

In both groups of columns, it can be noticed that the load bearing capacity was higher for NWAC elements than LWAC elements despite the similar compressive strengths of concrete. This tendency was more significant in columns with lower reinforcement ratios where concrete carried a relatively more substantial part of the total load. The displacement v_x_ increase during loading was also higher for elements made of LWAC with less longitudinal reinforcement (44 mm at maximum force for LWAC and 34 and 39 mm for NWAC elements), which is a result of the lower value of the modulus of elasticity for this kind of concrete. In stronger elements (Figure 11b), the displacement v_x_ at the maximum force was similar for both concrete types, about 55 mm. However, during loading, LWAC columns showed slightly lower stiffness.

It should be noted that the repeatability of the results was better for pairs with stronger reinforcement. The results are unclear for both cases when columns with #10 reinforcement were tested. The reason for this could be related to the stand, the elements or both. The recurrence of the divergences and their scale suggests that there could be an accidental eccentricity of force application in the second part of this research when columns labeled with number two were examined. On the other hand, another possible reason is the characteristics of materials and their contribution to load bearing. Steel exhibits greater stability in terms of the material properties, strength value and homogeneity in the elements. In contrast, concrete can be affected by the aggregate composition (especially in the case of LWAC) and compacting effectiveness of the concrete mixture. This may be why columns with stronger reinforcement have more consistent results.

The v_y_ displacement records (Table 6) show that the unintended deflection in the secondary plane was relatively high (CN-10-1) in only one column; in all other cases, it was negligible.

#### 3.1.3. Concrete and Steel Strain

Table 6 gives the results of compressive strain in concrete ε_c_ and tension reinforcement strains ε_s_ at the mid-height section of the columns and the corresponding curvature recorded for the maximum force F_u_. The values given in this table were calculated according to LVDT sensor readings.

**Table 6:** Summary of strain results.

Column		Strain Results at Maximum Force F_u_		
	v_xu_	ε_c_	ε_s_	κ
	m	‰	‰	1/m
CL-10-1	0.044	2.33	−2.49	0.044
CL-10-2	0.044	2.52	−3.05	0.051
CN-10-1	0.034	1.74	−3.21	0.045
CN-10-2	0.039	1.56	−5.17	0.062
CL-16-1	0.054	3.02	−3.02	0.056
CL-16-2	0.057	3.30	−2.92	0.058
CN-16-1	0.056	3.07	−4.14	0.067
CN-16-2	0.052	2.89	−3.07	0.056

**Figure 12:**
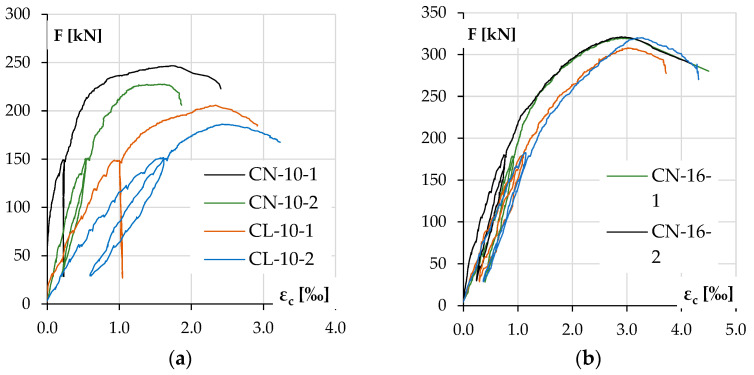
Experimental concrete compressive strain ε_c_ as a function of load F for the columns with the following: lower reinforcement ratios (**a**) and higher reinforcement ratios (**b**).

**Figure 13:**
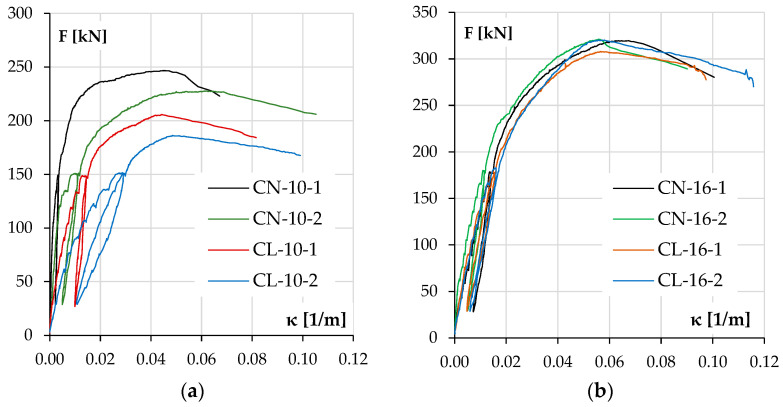
Experimental curvature at the mid-height of a column κ as a function of load F for the columns with the following: lower reinforcement ratios (**a**) and higher reinforcement ratios (**b**).

The concrete compressive strain ε_c_–load F responses for the tested columns are shown in Figure 12, and the curvature development during loading is shown in Figure 13. It should be noted that the curvature was calculated from the vertical LVDT sensor readings, while deflection was calculated from the horizontal ones, and the results are consistent.

When analyzing deformation (Table 6), the results for the corresponding elements can be considered similar to those expected. The trends are still evident, although they were slightly divergent for the low-reinforcement columns.

The differences in behavior between LWAC and NWAC elements are more evident for columns with low reinforcement. In this case, it can be seen that at the maximum force, LWAC columns showed higher concrete deformations on the compression side ε_c_ (Figure 12) but lower for tension steel ε_s_. Still, the curvature of the elements at the failure was similar (Table 6) for both types of concrete, although for LWAC columns, it was achieved at the lower maximum force F_u_ (compare Table 5). Still, the curvature recorded during the test at each force level was consistently higher for LWAC columns (Figure 13). It should be recalled that concrete was not crushed in either case in this group in the compression zone, so the ultimate strain ε_cu_ was not reached.

On the other hand, in the group of heavily reinforced columns, all elements behaved similarly regardless of the type of concrete. Table 7 shows that the concrete and steel strains and the curvature κ at the maximum force F_u_ are similar for all four elements. Figure 12 shows that the concrete compressive strain ε_c_ grew slightly faster for the LWAC columns, and the curvature κ was minimally higher (Figure 13). The only significant difference was noted at the time of failure, as in both columns with NWAC, there was the crushing of concrete on the compression side, which may suggest that the ultimate strain ε_cu_ was reached in this case.

### 3.2. Column Modeling

In the next stage of this research, a column model was created to simulate slender LWAC column behavior, compare it with their NWAC counterparts and validate it against experimental data.

#### 3.2.1. Numerical Model

A numerical model was created using OpenSees software (open-source, developed at University of California, Berkeley, US), which was developed to simulate the nonlinear response of structural elements.

A fiber-based model was used. In this case, materials have only uni-directional strength and stiffness, whose behavior is defined in terms of its stress–strain response. Fiber sections are assumed to remain plane throughout the analysis. In reinforced concrete structures, the fiber section is assembled with pre-defined concrete and steel materials. Strain compatibility between reinforcement and surrounding concrete is assumed. The sectional reactions under force and moment are in terms of axial strain at the mid-section and curvature.

**Figure 14:**
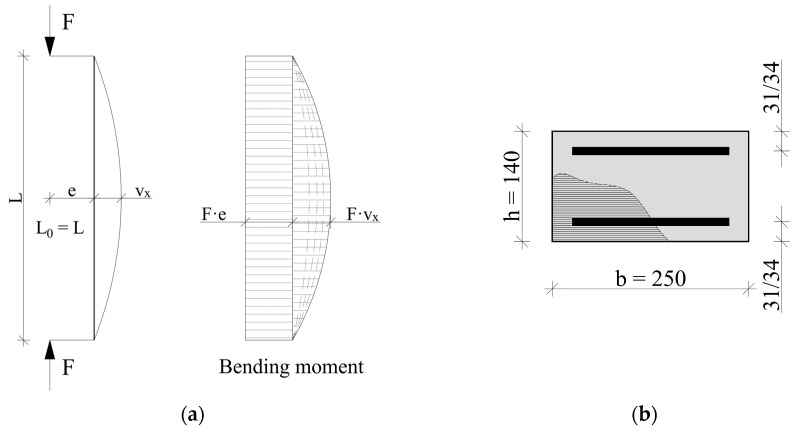
A static scheme of the column and bending moment diagram (**a**). A fiber cross-section of the element (**b**).

The column was modeled as a simply supported member subjected to a compressive force at a constant eccentricity e. The column nodes were constrained in the horizontal planes so that the maximum second-order moment would occur at the mid-height of the element. In the analysis of second-order effects, the Corotational Coordinate Transformation was used. The column was modeled with nonlinear beam–column elements with five integration points. The static scheme and the intended distribution of bending moments are shown in Figure 14a.

The cross-section of the column corresponds to the one designed in the experimental study. Following the fiber section approach assumptions, the concrete area was divided into 1 mm fibers according to Figure 14b. The column was reinforced symmetrically.

The length of the element L shown in Figure 14a was taken as 3 m, and the dimensions of the cross-section according to Figure 14b were 140 and 300 mm. The longitudinal reinforcement of the column was also assumed precisely as in the experiments consisting of 4 #10 or #16 bars. The effects of confinement from transverse reinforcement were ignored due to the relatively low ratio of this reinforcement. The eccentricity of the first-order moment e was 50 mm, the same as the one adopted in the experimental study.

The material characteristics for steel and concrete were defined using the UniaxialMaterial ElasticMultiLinear command.

**Figure 15:**
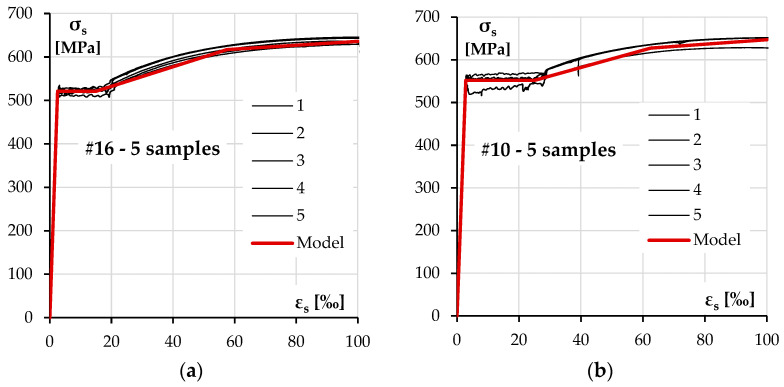
Stress–strain model of longitudinal reinforcement: #16 bars (**a**) and #10 bars (**b**).

The strength characteristic for the longitudinal reinforcing steel was defined according to the experimental results presented in the previous section. The diagrams used in the analysis are presented in Figure 15.

To simulate the results of this study, models of concrete were selected to provide a full nonlinear model of concrete, not only to determine the basic mechanical properties, and these could be applied to both NWC and LWAC. The breadth of the scope of this study and the details of the description of the models were also a consideration. Three nonlinear models for concrete under compression were considered in this study. These models were validated on numerous specimens made with different types of aggregate. The first one is the nonlinear model from Eurocode 2 (EC2) [18]. Since the previous analysis [30] raised some doubts about the estimated value of the elastic modulus and ultimate strain determined according to this model, two other models from the literature were applied. These were defined by Lim and Ozbakkaloglu in [31] (Lim 2014) and Tasnimi in [32] (Tasnimi 2004) and validated using experimental data for both LWAC and NWAC. The details of the models are presented in Table 7.

**Table 7:** Concrete models.

EC 2 [18]	Lim 2014 [31]	Tasnimi 2004 [32]
Model		
$\sigma_{c}=f_{c}\frac{\left(k\frac{\varepsilon_{c}}{\varepsilon_{0}}-{\left(\frac{\varepsilon_{c}}{\varepsilon_{0}}\right)}^{2}\right)}{1+\left(k-2\right)\frac{\varepsilon_{c}}{\varepsilon_{0}}}$ $k=1.05E_{c}\frac{\varepsilon_{0}}{f_{c}}$	$\sigma_{c}=\frac{f_{c}\left(\frac{\varepsilon_{c}}{\varepsilon_{0}}\right)r}{r\;-1+{\left(\frac{\varepsilon_{c}}{\varepsilon_{0}}\right)}^{r}}\;if\;0\leq \varepsilon_{c}\leq \varepsilon_{0}$ $r\;=\frac{E_{c}}{E_{c}\;-\frac{f_{c}}{\varepsilon_{0}}}$ $\sigma_{c}=f_{c}-\frac{f_{c}}{1+{\left(\frac{\varepsilon_{c}-\varepsilon_{0}}{{\varepsilon_{\mathrm{ci}}-\varepsilon }_{0}}\right)}^{r}}if\;\varepsilon_{c}>\varepsilon_{0}$	$\sigma_{c}=f_{c}\frac{n^{\mathrm{pq}}\frac{\varepsilon_{c}}{\varepsilon_{0}}}{{\left(\frac{\varepsilon_{c}}{\varepsilon_{0}}\right)}^{n^{\mathrm{pq}}}+n^{\mathrm{pq}}}$ $n^{\mathrm{pq}}=\frac{1}{1-\frac{f_{c}}{\varepsilon_{0}E_{c}}}\;\mathrm{for}\;\varepsilon_{c}\leq \varepsilon_{0}$ $n^{\mathrm{pq}}\;\mathrm{acc}\;\mathrm{to}\;$ [30] $\varepsilon_{c}>\varepsilon_{0}$
NWAC		
$E_{c}=22{\left(0.1f_{c}\right)}^{0.3}$ $\varepsilon_{0}=0.7{f_{c}}^{0.31}$ $\varepsilon_{u}=2.8+27{\left(0.01\left(98-f_{c}\right)\right)}^{4}$	$E_{c}=4400\sqrt{f_{c}}{\left(\frac{\rho }{2400}\right)}^{0.4}$ $\varepsilon_{0}=\frac{{f_{c}}^{0.225k_{d}}}{1000}$ $k_{d}={\left(\frac{2400}{\rho }\right)}^{0.45}$ $\varepsilon_{c.i}=10\varepsilon_{0}{f_{c}}^{-0.47}{\left(\frac{\rho }{2400}\right)}^{0.4}$	$E_{c}=60.03\ln{\left(\frac{f_{c}^{0.25}}{\rho^{0.2}}\right)}^{0.3}+20.575$ $\varepsilon_{0}=\left(65.39f_{c}^{0.35}-25.02\right)10^{-5}$
LWAC		
$E_{c}=22{\left(\frac{\rho }{2200}\right)}^{2}{\left(0.1f_{c}\right)}^{0.3}$ $\varepsilon_{0}=\frac{f_{c}}{E_{c}}$ $\varepsilon_{u}=\varepsilon_{0}$	$E_{c}=4400\sqrt{f_{c}}{\left(\frac{\rho }{2400}\right)}^{0.4}$ $\varepsilon_{0}=\frac{{f_{c}}^{0.225k_{d}}}{1000}$ $k_{d}={\left(\frac{2400}{\rho }\right)}^{0.45}$ $\varepsilon_{c.i}=10\varepsilon_{0}{f_{c}}^{-0.47}{\left(\frac{\rho }{2400}\right)}^{0.4}$	$E_{c}=2.1684f_{c}^{0.535}$ $\varepsilon_{0}=\left(65.57f_{c}^{0.44}-6.748\right)10^{-5}$

Note: the subscripts and superscripts were made consistent for each model, and the symbols are explained below: $\varepsilon_{c}$—concrete strain [–]; $\varepsilon_{0}$—concrete strain at the peak stress [–]; $\varepsilon_{u}$—ultimate concrete strain [–]; $\sigma_{c}$—concrete compressive stress [MPa]; $f_{c}$—concrete compressive strength [MPa]; $E_{c}$—modulus of elasticity [GPa]; ρ—density of concrete [kg/m^3^].

**Figure 16:**
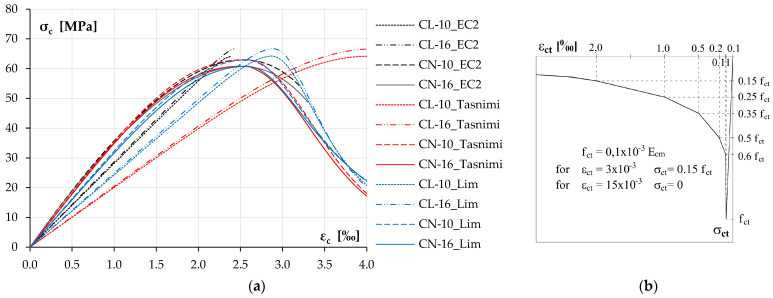
Material models for concrete under compression (**a**). Material model for concrete under tension (**b**).

The material models for concrete under compression for each element type analyzed in the research created based on the above formulas and used in the calculations are shown in Figure 16a. After analyzing the deformation results of the tests, the value of $\varepsilon_{u}$ in the analyzed models was limited to 4‰, which covers the range of deformations obtained in the tests. This value could not even be achieved in some cases, as the model assumed lower limitations (EC2—black lines).

For the tensile zone, the model developed in [33] was adopted, taking into account tension stiffening with the smeared cracking effect. The idea of the model is shown in Figure 16b.

The concrete properties determined according to the adopted models, taking into account the experimentally obtained values of compressive and tensile strengths, are summarized in Table 8.

It should be noted that several significant differences can be observed when comparing the calculated concrete parameters with those obtained experimentally (Table 3). In all cases, the values of the elastic modulus calculated according to the EC2 procedure turned out to be overestimated compared to those measured during testing. The most accurate estimate of the elastic modulus for LWAC was obtained using the Tasnimi 2004 model, while for NWAC, it was obtained using the Lim 2014 model.

#### 3.2.2. Validation of Numerical Models Against Experimental Data

Considering the above assumptions, calculations of columns with parameters reflecting those adopted in this research were carried out, followed by the verification of the adopted model.

The analysis results are presented as diagrams of the horizontal displacement v_x_ of the column at half its height to the acting axial force F. The diagrams show the experimental curves (black lines) for each pair of identical columns and the plot obtained from calculations for the three models.

**Figure 17:**
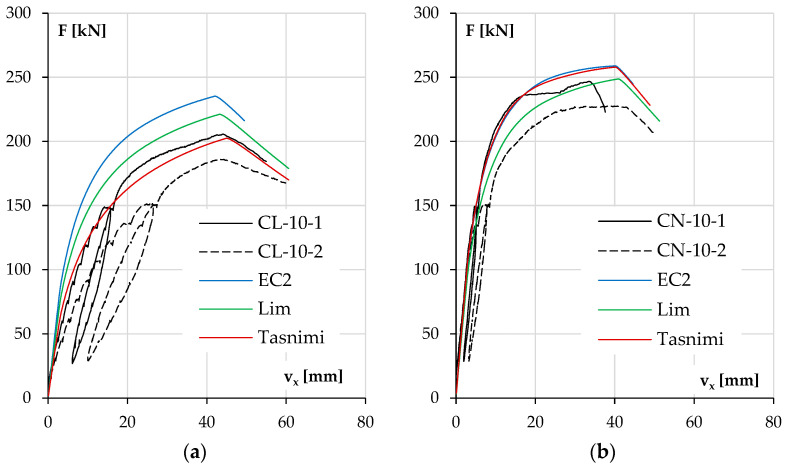
The experimental and calculated displacement v_x_ results as a function of load F for the columns with lower reinforcement ratios and made of LWAC (**a**) and NWAC (**b**).

**Figure 18:**
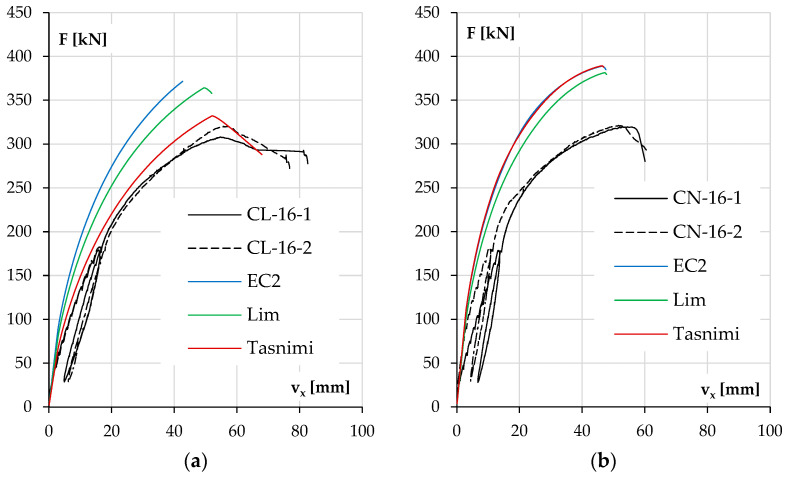
The experimental and calculated displacement v_x_ results as a function of load F for the columns with higher reinforcement ratios and made of LWAC (**a**) and NWAC (**b**).

Analyzing the results, it can be seen that the main parameter determining the consistency of the results was, as expected, the elastic modulus of concrete. As mentioned earlier in the case of LWAC, the elastic modulus determined in the tests was the same as the one calculated according to the Tasnimi 2004 model, which is reflected in the curves shown in Figure 17a and Figure 18a. The closest estimate of the elastic modulus for NWAC was obtained from the Lim 2014 model, and it had the lowest calculated value. However, it was still 7% higher than that obtained from testing cylindrical specimens. These results are presented in Figure 17b and Figure 18b.

In both low-reinforcement columns (Figure 17a,b), the experimental results are affected by the aforementioned additional unintended eccentricity in the second series of columns, so the following conclusions will apply to the columns of the first series (CL-10-1, CN-10-1). The consequences of measurement difficulties are discussed more fully in the next point. As mentioned above, the behavior of the CL-10-1 column is best approximated by calculations using the Tasnimi 2004 model and the CN-10-1 column with the Lim 2014 model. For the LWAC element, both the load bearing capacity and the deflection at the maximum force are very close (differences of 2% and 4%, respectively). The other two models in this case, due to the much higher value of the elastic modulus, had the effects of underestimating the displacement at each load level and thus the second-order effects, which resulted in the overestimation of the load carrying capacity. In the case of the CN-10-1 column, the load bearing capacity was found to be most accurately estimated using the Lim 2014 model; the difference in the value of maximum forces was 5%. This model also allowed for the most accurate estimation of the elastic modulus of concrete (see Table 3 and Table 8). A representative comparison of deflections is impossible due to the appearance of displacement in the secondary direction above a force of 230 kN (see Table 5).

In the case of LWAC columns with higher ratios of reinforcement (Figure 18a), the red line referring to the Tasnimi 2004 model also represents the element’s behavior very well. The calculated load capacity was overestimated by 4% and 8% compared to the experimental values. The theoretical deflection (53 mm) at the maximum force was slightly lower than that obtained in the test (57, 54 mm)—differences of 7% and 2%. The results for the other two models differed analogically from those of the LWAC columns with #10 bars.

The results for the heavily reinforced NWAC columns were questionable. The experimental results for both elements were very consistent, and as mentioned above, the elastic modulus estimated using the Lim 2014 model differed only slightly from that obtained in the tests. Yet, the calculated load capacity results far exceeded the experimental values recorded in the test, while the displacements were underestimated. The closest value of the maximum force was calculated using the model mentioned above (381 kN) and exceeded the load capacity from the tests (about 320 kN) by 19%. This problem will be discussed in more detail in the next point.

#### 3.2.3. The Effects of Technical Defects on the Test Results

After analyzing the test results and the corresponding analytical curves, it can be concluded that it is highly likely that technical inaccuracies affected the test results. Among the most significant issues are the accuracy of force application (eccentricity) and the stiffness of the entire stand, which may have affected the deflection of the elements.

**Figure 19:**
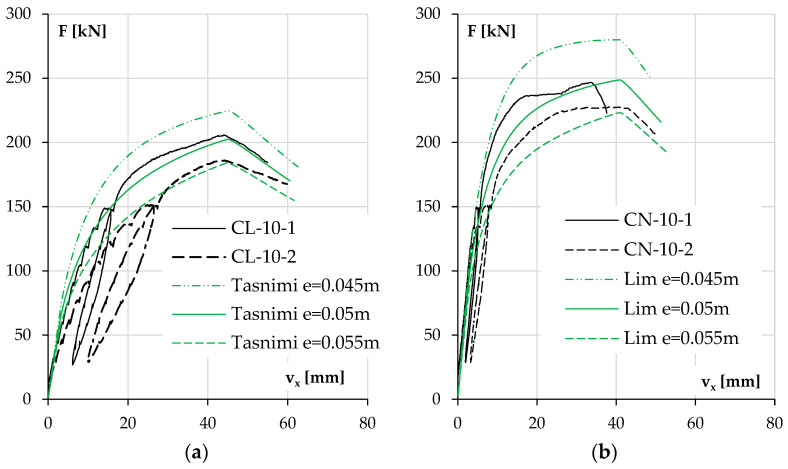
For different force eccentricities, the experimental and calculated results of displacement v_x_ as a function of load F for columns with low reinforcement ratios and made of LWAC (**a**) and NWAC (**b**).

**Figure 20:**
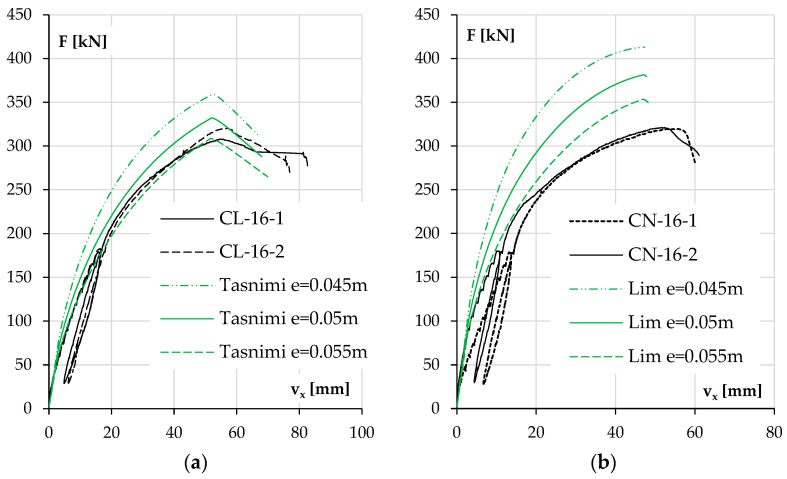
For different force eccentricities, the experimental and calculated results of displacement v_x_ as a function of load F for columns with high reinforcement ratios and made of LWAC (**a**) and NWAC (**b**).

First, the possibility of an unintended eccentricity and its impact on the results were analyzed. An unintended eccentricity of 5 mm was assumed. The results for each pair of elements are shown in Figure 19 and Figure 20. Only one concrete model was included in these calculations, the one that best represented the behavior of the columns in the previous section, mainly due to the accurately estimated elastic modulus of concrete.

During the conducted tests, columns CL-10-1 and CN-10-1 were tested first; then, due to the deformation of the head plate, it was repaired. This step was justified, but it appeared to have caused an unintended eccentricity in the following columns, as shown in Figure 19 and Figure 20. This effect is also evident in the difference that appeared in the results between the twin columns in Figure 19a,b.

A closer investigation of the diagram in Figure 20b reveals a sudden slope in the experimental curves for these two elements as their stiffness above a force of 230 kN decreased much faster than predicted by the adopted model.

**Figure 21:**
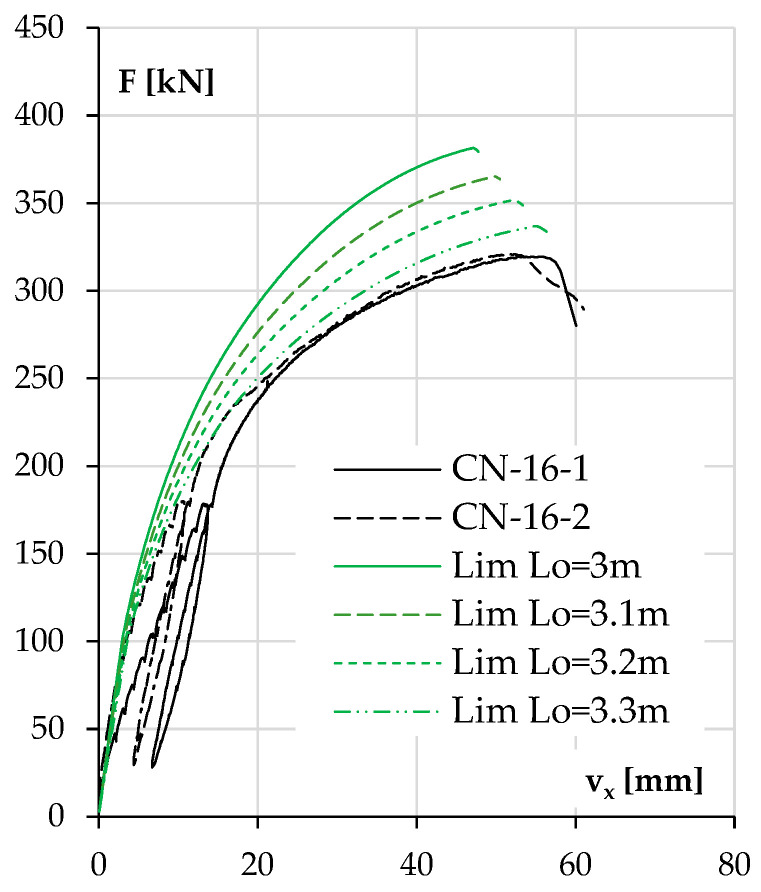
The experimental and calculated displacement v_x_ results as a function of load F for the NWAC columns with high reinforcement ratios obtained taking different buckling lengths.

It should be noted that the results of the two twin elements (CN-16-1 and CN-16-2) were very consistent, which excludes execution errors of a random nature. The concrete mix used was the same as that in the NWAC columns with #10 bars. However, these two elements had the highest stiffness among the columns included in the research plan, so in this particular case, the reason for underperformance could be the deformation of the test stand, causing additional displacements, even if this were not observed in the case of the other elements. If the deformation of the test stand was associated with the dislocation of the theoretical non-moving nodes of the element, then this could be depicted as an exaggerated buckling length. An analysis of such a situation is shown in Figure 21.

Figure 21 shows the behavior of the columns, with an increasing theoretical buckling length as a result of the deformation of the stand. The calculations used a nominal force eccentricity of 0.05 m and the Lim 2014 model for concrete. It can be seen that a change in buckling length resulted in an increase in theoretical deflection at the maximum force, in contrast to the diagrams shown in Figure 19 and Figure 20, where a change in eccentricity resulted in a change in the maximum force but not in the corresponding deflection. This confirms that the results for the two columns shown in Figure 21 were not only affected by the unintentional eccentricity.

## 4. Discussion

This paper presents an experimental study of slender LWAC and NWAC columns. This research aimed to compare the behavior of elements sensitive to second-order effects, assuming that they will be made of concretes of similar strength but different types. The main differences considered in this case are the significantly different elastic modulus and ultimate strain.

Studies of this type are difficult to carry out due to the technical difficulty of eliminating unwanted external effects, making it impossible to isolate the factors under study. Some of these difficulties could not be avoided, so the test had an unwanted accidental eccentricity (see Figure 20). In the case of the strongest pair of columns (CN-16-1, CN-16-2), the deformation of the stand could overestimate the increasing deflection and, at the same time, underestimate the recorded load capacity of the elements (see Figure 21). When analyzing the results of the tests presented, these facts have to be taken into account. Even facing the above difficulties, it was possible to draw some important conclusions.

As expected, this study showed that for slender columns, the type of concrete significantly affects the behavior of the elements under load. All columns showed typical buckling at failure. However, elements from LWAC with a lower modulus of elasticity exhibited higher displacements throughout the loading process and ultimately achieved lower maximum force values. For columns reinforced with #10 bars, columns with LWAC (v_xu_ = 0.044 m, 0.044 m) had an average 18% lower load bearing capacity than elements with NWAC (v_xu_ = 0.034 m, 0.039 m), while with #16 reinforcement, the difference was lower, as it was only 2%. There was no clear trend when it came to deflection at the maximum force (about 0.055 m for each element), but in this case, according to the comments above, the displacement results for NWAC columns may be overestimated. This means that stronger reinforcement would have to be used in LWAC columns to achieve the same load bearing capacity.

A further part of this work was devoted to an attempt to model concrete columns using different concrete models in the compression zone. The analysis showed that the key to modeling the realistic behavior of columns is the correct assessment of its stiffness, which is directly related to the elastic modulus value. The modulus was calculated according to the models’ assumptions in the calculations presented. A comparison of the results showed that the best model for LWAC columns was the Tasnimi 2004 model, while for NWAC columns, it was the Lim 2014 model, which is because, in this case, the modulus values were the closest to the experimental ones. The model adopted in [19] overestimated the stiffness in all cases and, thus, the load bearing capacity of the elements. Moreover, the maximum strain calculated according to [19] for LWAC in this case was about 2.4‰ (Table 8), which turned out to be accurate in the case of columns reinforced with #10 bars (Figure 17a, Table 6) but somewhat conservative in the case of columns with #16 bars (Figure 18a, Table 6), where the average strain was 2.96 ‰, as can be seen in the graph shown.

## 5. Conclusions

The experimental program showed that the design of slender columns requires the particular consideration of the mechanical properties of LWAC beyond its strength. Particularly important is determining the realistic value of the modulus of elasticity.

This study confirmed the higher deformation of LWAC columns, which, especially with low reinforcement, leads to increased second-order effects. This fact needs to be taken into account in design procedures through the precisely calculated stiffness of elements.

Of concern is the fact that the behavior of the column modeled by the commonly used model from [18] led to the overestimation of the load carrying capacity. Also, models defined experimentally in numerous tests of actual specimens proved to be an uncertain tool for evaluating the load capacity of slender columns. With such a scattering of results at this stage, it must be concluded that there is no certain model that we can use to predict the exact load bearing capacity of this type of element. This issue certainly requires deeper analysis not only in terms of nonlinear element studies but also in terms of design models, which is the future intention of the authors of this paper.

## Figures and Tables

**Table 1:** The details of the columns.

Column	Geometry			Reinforcement		Type of Concrete
	L	h	b	A_s_ (No 1)	A_sw_ (No 2)	
	mm	mm	mm	mm	mm	
CL-10-1	3000	140	250	4#10	#8 at 150/50	LWAC
CL-10-2						LWAC
CN-10-1						NWAC
CN-10-2						NWAC
CL-16-1				4#16		LWAC
CL-16-2						LWAC
CN-16-1						NWAC
CN-16-2						NWAC

**Table 2:** The mix proportions of LWAC and NWAC (per 1 m^3^).

Type of Concrete	Aggregate		CEM I 42.5 R	Water	w/c	Additives
	Course	Fine				
	kg	kg	kg	kg		
LWAC	752	410	480	220	0.46	Sika Fume–41 kg
NWAC	1012	736	410	170	0.41	Fly ash–30 kg VISCOCRETE 3088 M (SIKA)–2.66 kg BV 12 (SIKA)–1.02 kg

**Table 3:** Concrete properties.

Column	Age Days	Concrete Properties on the Day of the Test ^1^				
		r	f_c_	E_c_	f_c,cube_	f_ct,sp_
		kg/m^3^	MPa	GPa	MPa	kg/m^3^
CL-10-1	112	1860	62.4; 59.8 (61.1)	19.1; 19.2 (19.2)	68.9	2.5
CL-10-2	128	1820	66.3; 68.2 (67.3)	18.6; 18.9 (18.8)	70.5	3.0
CL-16-1	124	1861	65.9; 66.5 (66.2)	19.2; 20.0 (19.6)	63.6	3.5
CL-16-2	135	1831	67.2; 67.0 (67.1)	21.8; 21.4 (21.6)	69.2	2.4
CN-10-1	98	2289	62.7; 63.2; 62.3 (62.7)	31.2; 30.5; 30.5 (30.7)	65.1	5.3; 4.4 (4.9)
CN-10-2	118	2276	64.3; 60.5; 64.3 (63.0)	31.4; 31.0; 31.2 (31.2)	68.5	4.7; 4.8 (4.8)
CN-16-1	106	2292	59.9; 56.6; 61.3 (59.3)	30.7; 28.6; 30.6 (30.0)	73.9; 71.1 (72.5)	4.7; 4.8 (4.8)
CN-16-2	127	2270	61.1; 63.4; 62.0 (62.2)	32.3; 32.5; 32.1 (32.3)	66.8; 65.4 (66.1)	5.3; 5.5 (5.4)

^1^ The values for each tested sample with the average value in parentheses.

**Table 4:** The properties of the steel reinforcing bars used.

Diameter	Net Area	Yield Strength f_y_	Tensile Strength f_u_	Yield Strain ε_y_	Ultimate Strain ε_u_	Modulus of Elasticity E_s_
mm	mm ^2^	MPa	MPa	‰	‰	GPa
8	51.4	534 (5/0.7%) ^1^	661 (5/0.2%)	-	103 (5/1.3%)	197 (5/2.1%)
10	78.4	552 (5/2.1%)	647 (5/1.5%)	2.8 (5/1.7%)	102 (5/4.1%)	195 (5/2.5%)
16	199.6	521 (5/1.1%)	636 (5/1.1%)	2.5 (5/1.4%)	103 (5/11.5%)	208 (5/1.5%)

^1^ Values in parentheses describe the number of samples and the corresponding coefficient of variation characterizing the test results. ^2^ The average net area calculated for the given number of samples.

**Table 5:** Summary of test results.

Column		Final Results		
	f_c_/f_lc_^1^	F_u_	v_xu_	v_yu_
	MPa	kN	m	mm
CL-10-1	61.1	206	0.044	0.000
CL-10-2	67.3	186	0.044	0.001 ^2^
CN-10-1	62.7	247	0.034	0.012
CN-10-2	63.0	228	0.039	−0.001
CL-16-1	66.2	308	0.054	0.005
CL-16-2	67.1	320	0.057	0.001
CN-16-1	59.3	320	0.056	0.001
CN-16-2	62.2	321	0.052	0.000

^1^ Average value according to Table 4. ^2^ Only for one level of sensors, due to malfunction.

**Table 8:** Concrete properties.

Column Type	Experimental Values ^1^		Lim 2014 [31] Model		Tasnimi 2004 [32] Model		EC 2 Model		
	r	f_c_	E_c_	$\varepsilon_{0}$	E_c_	$\varepsilon_{0}$	E_c_	$\varepsilon_{0}$	$\varepsilon_{u}$
	kg/m^3^	MPa	Gpa	‰	Gpa	‰	Gpa	‰	‰
CL-10	1840	64.2	24.3	2.87	20.1	4.03	26.9	2.39	2.39
CL-16	1846	66.7	24.9	2.90	20.5	4.09	27.4	2.44	2.44
CN-10	2283	63.0	32.6	2.59	36.3	2.54	38.2	2.53	3.21
CN-16	2281	60.8	32.0	2.57	35.8	2.51	37.8	2.50	3.32

^1^ Average experimental values calculated for two columns of the same type.

## Data Availability

The original contributions presented in this study are included in the article. Further inquiries can be directed to the corresponding author.
